# Willingness to Pay (WTP) for Newer Treatment Options for Diabetes: A Study Among Patients at a Tertiary Care Centre

**DOI:** 10.7759/cureus.56103

**Published:** 2024-03-13

**Authors:** Shrutangi Vaidya, Shubham Atal, Rajnish Joshi

**Affiliations:** 1 Endocrinology and Diabetes, All India Institute of Medical Sciences, Bhopal, Bhopal, IND; 2 Pharmacology, All India Institute of Medical Sciences, Bhopal, Bhopal, IND; 3 Internal Medicine, All India Institute of Medical Sciences, Bhopal, Bhopal, IND

**Keywords:** icodec, type 1 and type 2 diabetes mellitus, su (1): shared-decision making, willingness-to-pay threshold, semaglutide, rybelsus, pharmacoeconomics, drug costs

## Abstract

Introduction

Type 2 diabetes mellitus (T2DM) poses a substantial burden globally and particularly in India, affecting health, finances, and overall quality of life. The management of this condition relies on lifestyle modifications and advanced pharmacological interventions, with emerging drugs showing promise in areas such as administration, side effects, efficacy, and cardiovascular benefits. However, their market penetration is hindered by high costs. Understanding the target population's expectations and willingness to pay (WTP) for these drugs is crucial. WTP, a key concept in behavioral science, reflects the maximum price consumers are willing to pay for a product, aiding in healthcare cost-effectiveness evaluations. Despite its relevance, only one WTP study has been conducted in the Indian context for diabetes. This study explores WTP for two novel drugs: oral semaglutide and icodec (weekly insulin).

Material and methods

This observational study, conducted in a diabetes specialty clinic and telemedicine facility in All India Institute of Medical Sciences, Bhopal, India, involved adults (18-80 years) diagnosed with T2DM. Data collection adhered to ethical guidelines, and participants provided written informed consent. Face-to-face interviews were employed to gather socio-economic, demographic, and medical details. Participants estimated their WTP for oral semaglutide and weekly insulin, considering reference ranges for existing antidiabetic treatments. Statistical analyses, including t-tests and analysis of variance, explored sociodemographic and clinical factors influencing WTP.

Results

Of 105 approached patients, 87 (74.3%) participated. The majority were males (55.2%) with an average age of 57.2 years. The average WTP for oral semaglutide was INR 9.35±5.66 per pill, significantly lower than its market price (INR 315). For weekly insulin (icodec), the WTP was INR 157.25±112.60 per dose. Subgroup analyses revealed no significant correlations based on sociodemographic or clinical parameters.

Conclusion

This study demonstrated the feasibility of WTP assessments in an Indian outpatient setting, revealing a substantial cost disparity between patients' WTP for oral semaglutide and its market price. The findings underscore the importance of considering WTP in introducing new diabetes medications in India, offering valuable insights for healthcare decision-makers and developers.

## Introduction

The burden of type 2 diabetes mellitus (T2DM) both in India and worldwide is significant, impacting health, finances, and overall quality of life [1]. Lifestyle modifications and advanced pharmacological interventions are key to managing this disorder, with new drugs expected to outperform existing treatments in various aspects such as administration, side effects, efficacy, and cardiovascular benefits [2].

Despite their superiority, these novel drugs often struggle to gain traction in the market due to their higher costs. Therefore, understanding the target population's expectations and willingness to pay (WTP) for these drugs is crucial. Such insight will shed light on the perceived value of the drugs' qualities and help developers comprehend the preferences and expectations of individuals with diabetes.

In behavioral science, WTP represents the maximum price at which consumers would purchase a product. It is a vital concept in evaluating the cost-effectiveness of healthcare interventions, especially when resources are limited. WTP studies provide valuable information on drug value when there is no existing market or when market prices do not reflect actual costs [3]. This method aids decision-makers in healthcare and health promotion priorities, capturing the preferences of those directly benefiting from proposed treatments. Although most economists consider WTP an acceptable method when aligned with economic analysis goals, only one WTP study has been conducted in the Indian setting for diabetes [4].

For assessing WTP for newer antidiabetic treatments, we have explored two novel drugs - oral semaglutide and icodec (weekly insulin).

Oral semaglutide represents a groundbreaking advancement in diabetes management as the first oral GLP-1 receptor agonist available in India. Its convenient oral formulation offers a viable alternative to injectable medications, addressing patient and healthcare provider reluctance. With demonstrated efficacy in weight loss and HbA1C reduction, alongside additional cardiovascular benefits, oral semaglutide holds promise for improving outcomes in patients with type 2 diabetes [5].

Weekly insulin injection (icodec) stands out as a novel approach to insulin therapy, offering the convenience of once-weekly administration compared to traditional daily injections. This regimen aims to enhance treatment adherence and patient acceptance, potentially overcoming barriers associated with frequent injections. With notable reductions in HbA1C and minimal adverse effects, weekly insulin injection presents a promising option for simplifying insulin therapy and improving patient outcomes in type 2 diabetes management. It has demonstrated significantly better efficacy compared to once-daily insulin glargine U100 in patients with type 2 diabetes [6] but is not yet available in India.

Employing the WTP experiment for these drugs, both of which represent innovative developments in diabetes treatment, helps assess whether the increased costs of these new drugs align with the expectations and affordability of persons with diabetes in India. By gauging the value attributed to these medications, this study contributes valuable insights to improve healthcare decision-making and resource allocation.

## Materials and methods

This observational study was conducted at a diabetes specialty clinic and telemedicine facility for outpatients in All India Institute of Medical Sciences, Bhopal, India. Data was collected in July and August 2022 using convenience sampling following the ICMR's National ethical guidelines for biomedical and health research on human participants (2017) after obtaining permission from the Institutional Human Ethics Committee - Post Graduate Research (IHEC-PGR/2022/STS-ICMR/7).

The study population consisted of willing adults (18-80 years old) of both sexes, diagnosed with T2DM, and currently on any allopathic antidiabetic therapy, including metformin. Participants with known psychiatric/neurological conditions affecting cognition or comprehension were excluded.

Participants were approached individually and after obtaining written informed consent, face-to-face interviews were conducted to ensure survey reliability and validity. To minimize interviewer bias, all interviews were conducted by a single interviewer (S.V.), ensuring consistency in the information provided to participants. This information was limited to what was available in our drug information leaflets. Socio-demographic and clinical details were recorded using a survey form. Leaflets with all relevant information regarding oral semaglutide and weekly insulin injections, along with costs per lowest dose of current commonly prescribed drugs were provided as reference (see Table 3 in Appendices) to the participants, followed by verbal explanations for the same.

Participants were asked to estimate the price they would be willing to pay for these drugs (per lowest dose). Data was tabulated in the IBM SPSS Statistics for Windows, Version 25 (Released 2017; IBM Corp., Armonk, New York, United States), and basic descriptive statistics were employed to generate study results. After confirming the normality of our data through the Shapiro-Wilk test (p=0.7) and the homogeneity of variances across groups via Levene's test (p=1.3), we employed unpaired t-tests and analysis of variance (ANOVA) to assess whether participants with specific sociodemographic or clinical profiles (e.g., presence or absence of comorbidities such as hypertension or dyslipidemia, duration since initiation of T2DM treatment, glycemic status control) exhibited significantly different WTP costs.

## Results

Among 105 patients approached for the WTP experiment, 87 (74.3% participation rate) provided estimates for the cost they were willing to pay for oral semaglutide and weekly insulin injection. About 18 participants were excluded: 8 declined consent and 10 could not provide estimates due to language or comprehension issues.

Participant profile

Among the 87 analyzed participants, 55.2% were males. The average age of participants was 57.2±11.4 years, the average duration since diagnosis of diabetes was 11.3±5.6 years, while 64.4% (n=56) of them had comorbidities (hypertension, dyslipidemia, etc.) along with diabetes, and most belonged to the lower middle (28, 43.1%) or upper middle (24, 36.9%) socioeconomic classes according to modified Kuppuswamy scale. Demographic and clinical characteristics of the participants are presented in Table 1.

**Table 1:** Socio-demographic profile of participants BMI: Body mass index; T2DM: Type 2 diabetes mellitus; HTN: Hypertension

S.No.	Characteristics	Mean/N (%)
1.	Gender (n=87)	
	Male	48(55.2)
	Female	39(44.8)
2.	Age (in years) (n=87)	57.2±11.4
3.	BMI (in kg/m^2^) (n=63)	25.6±3.9
	Normal	34 (53.4)
	Overweight/obese	29 (46.6)
4.	HbA1C (n=65)	
	Controlled (<7%)	27 (41.5)
	Uncontrolled (≥7%)	38 (58.5)
5.	Education (n=65)	
	Middle school	3 (4.6)
	High school	13 (20)
	Intermediate/Diploma	32 (49.2)
	Graduate	17 (26.1)
6.	Occupation (n=65)	
	Semiskilled worker	3 (4.6)
	Skilled worker	9 (13.8)
	Clerical/shop/farm	11 (16.9)
	Semi-professional	34 (52.3)
	Professional	5 (7.6)
	Semiskilled worker	3 (4.6)
7.	Monthly income (n=65)	
	INR 6,175-18,496	20 (30.7)
	INR 18,497-30,830	17 (26.1)
	INR 30,831-46,128	15 (23.1)
	INR 46,129-61,662	9 (13.8)
	INR 61,663-1,23,321	3 (4.6)
	≥INR 1,23,322	3 (4.6)
8.	Socioeconomic status (n=65)	
	Upper	3 (4.6)
	Upper middle	24 (36.9)
	Lower middle	28 (43.1)
	Upper lower	10 (15.4)
9.	Average time since diagnosis of T2DM (in years) (n=62)	11.3±5.6
10.	Comorbidities (HTN, dyslipidemia) (n=56)	
	With	36 (64.4)
	Without	20 (35.6)
11.	Complications (n=56)	
	With	31 (55.4)
	Without	25 (44.6)

The average estimated cost for one tablet of oral semaglutide that patients were willing to pay was INR 9.35±5.66, with a median being INR 10, and for one dose of weekly insulin (icodec) was INR 157.25±112.601 with a median of INR 150. On conducting subgroup analysis based on different sociodemographic and clinical parameters, for oral semaglutide and insulin icodec separately, no significant differences were found between the estimated costs of the different subgroups, as shown in Table 2.

**Table 2:** Sub-group analysis for estimated cost of oral semaglutide and insulin icodec *T2DM: Type 2 diabetes mellitus; INR: Indian rupee; ANOVA: Analysis of variance

S.No.	Characteristic	n	Estimated cost in INR mean±SD	t-statistic, p-value (unpaired t-test)	Estimated cost in INR mean±SD	t-statistic, p-value (unpaired t-test)
			Oral semaglutide		Insulin icodec	
1.	Gender			t=1.38, p=0.89		t=0.43, p=0.82
	Male	48	9±5.97		162.5±82.8	
	Female	37	9±5.86		143.7±80.1	
2.	Glycemic status			t=-0.71, p=0.51		t=-0.27, p=0.79
	Controlled (<7% HbA1C)	35	8.9±5.75		183±101.3	
	Uncontrolled (≥7% HbA1C)	52	9.1±5.4		167±91.5	
3.	Years since T2DM* diagnosis			t=-0.68, p=0.11		t=-1.57, p=0.63
	<10 years	28	9.4±5.82		154.2±93.7	
	≥10 years	57	9±5.25		173.4±82.3	
4.	Comorbidities			t=-1.87, p=0.54		t=-0.49, p=0.43
	Present	50	8.9±5.81		166.8±59.4	
	Absent	37	9.3±4.9		159.6±86.4	
				ANOVA p-value		ANOVA p-value
5.	Socioeconomic status			p=0.23		p=0.36
	Upper	15	9±4.5		176.8±67.9	
	Upper middle	24	9.8±5.2		150±45.9	
	Lower middle	28	9.3±4.8		168.7±70.1	
	Lower	20	9.3±4.7		157.3±88.1	

## Discussion

The "WTP" model is widely accepted in pharmacoeconomics and has been extensively used in international studies [7-9], to determine preferred drug attributes and patient payoffs for different features. However, limited WTP studies have been conducted in the Indian healthcare setting, particularly for diabetes mellitus [4].

Our study revealed that the concept of WTP was well understood by the target population, and the survey was relatively easy to conduct during outpatient department rush hours in Indian settings, requiring only 10-15 minutes per patient. Patients encountered some difficulties in determining the cost of insulin icodec, struggling to decide whether to use the weekly cost or the cost of a single shot of currently available insulin as a reference, which we navigated by providing the weekly costs of commonly prescribed insulin-based regimens.

Several studies [10,11] have emphasized attributes such as the oral route of administration and reduced frequency as preferable for patients. This aligns with the feedback we received from patients, emphasizing the positive aspects of the oral route of administration for semaglutide and the reduced frequency of injectable insulin administration.

The main contributions of this WTP study include enabling the quantification of estimated costs for new drugs in the market while adhering to good research practices. The sociodemographic data indicates the representativeness of the patient population in central India, offering an apt estimate of the affordability of such drugs. An interesting finding is that despite differences in the sociodemographic and clinical profiles of participants, the uniformity in the perceived value and WTP for these drugs suggests that if the actual costs of these drugs significantly differ from the estimated costs, it may pose challenges for their acceptance and popularity in the diverse Indian market [12].

Notably, our study exposed a significant disparity between the expected cost of oral semaglutide (average INR 9.35±5.66 per pill) and its actual market price (INR 315 per pill as announced by Novo Nordisk) [13]. The current costs of insulin-based regimens (10U glargine, 20U glargine, 10U glargine, and 5,5 Lispro) range from INR 250 to more than INR 500 per week, which are considerably higher than the median estimated cost of INR 150 of insulin icodec [14,15].

However, we acknowledge certain limitations of our study. While economic variables were not directly analyzed in this study, future research could explore the relationship between patient preferences, WTP, and economic factors such as income, affordability, and healthcare access to provide a more comprehensive understanding of medication pricing and affordability in the Indian healthcare context. Owing to logistical constraints, our study was confined to a single tertiary care center, and our relatively small sample size, acquired through convenience sampling, may have impacted the generalizability of our findings. We suggest that future studies be conducted in diverse settings, employing robust sampling techniques such as random sampling on a larger population, to enhance the validity of our results.

## Conclusions

Our study revealed that the concept of WTP was well understood by the target population, and the survey was relatively easy to conduct during OPD rush hours in Indian settings, requiring only 10-15 minutes per patient. Notably, our study exposed a significant disparity between the expected cost of oral semaglutide (average INR 9.35±5.66 per pill) and its actual market price (INR 315 per pill as announced by Novo Nordisk).

The study, provided insights into the patients’ estimated price of insulin icodec while identifying significant cost discrepancies for oral semaglutide. It also highlighted the importance of WTP assessments for new drugs in the Indian healthcare context.

## Appendices

Information leaflet

Oral Semaglutide

This medicine belongs to a class of medications called GLP-1 Agonists. They are well-established treatment options for the treatment of T2DM. Despite the health benefits and guideline recommendations of these drugs, both patients and healthcare professionals are reluctant to initiate these drugs. This is likely attributable to the fact that until recently, they could be administered as injections only. With the advent of oral semaglutide in the market, these newer and efficacious drugs can now be administered in tablet form now.

Frequency of administration is once daily; patients must wait at least 30 minutes before taking any other oral medication. Oral semaglutide has been shown to cause 14 mg - 3.7 kg of weight loss; better than other GLP-1 analogs (except injectable semaglutide). Has been shown to have a 1.8% decrease in HbA1C (after six months of treatment). Other benefits include a modest reduction in blood pressure, reduced risk of atherosclerotic cardiovascular disease, stage 3 chronic kidney disease, or heart failure with reduced ejection. Adverse effects include nausea and diarrhea (mild to moderate in severity), rarely pancreatitis. Hypoglycemic events are rare.

Weekly Insulin Injection

Instead of daily injections, once-a-week insulin injections may help increase adherence and acceptance. It is administered as a subcutaneous injection. The frequency of administration once weekly has been shown to have a 1.33% reduction in HbA1C (after six months). Adverse effects are similar to those caused by daily insulin analogs - little weight gain, low risk of injection-site reactions, and hypersensitivity reactions. Hypoglycemic events might occur with sporadic exercise or with skipped meals but in general, should not be a major issue.

The average costs of the currently available drugs are shown in Table 3.

**Table 3:** Average costs and route of administration of currently available drugs

Drug	Dose	Route of administration	Cost (Rs)
Metformin	500 mg	Oral	1.52
Regular insulin	10 U	Injectable	3.74
Insulin degludec	10 U	Injectable	61.67
Dapagliflozin	10 mg	Oral	15.2
Glimepiride	1 mg	Oral	2.6
Teneligliptin	20 mg	Oral	9.9
Glargine	10 U	Injectable	25.44
Pioglitazone	15 mg	Oral	3.23
Liraglutide	1.2 mg	Injectable	305.1
